# Cytoskeleton imaging of colorectal and lung cancer spheroids using light sheet microscopy

**DOI:** 10.1038/s44276-025-00144-3

**Published:** 2025-06-18

**Authors:** Sonia Prado-López, Massih Foroughipour, Klaus Becker, Seyed Meraaj Foroughipour, Lukas Weber, Heinz Wanzenboeck, Nika Sarem, Saiedeh Saghafi

**Affiliations:** 1 Research Unit of Nanoelectronic Devices, Institute of Solid State Electronics, Faculty of Electrical Engineering and Information Technology, TU Wien, Vienna, Austria; 2 Meso-Aspheric Optics & LSFM Group, Institute of Solid State Electronics, Faculty of Electrical Engineering and Information Technology, TU Wien, Vienna, Austria; 3 Medical University of Graz, Graz, Austria

## Abstract

**Background:**

Three dimensional tumoral models are essential to study cancer biology as they better mimic the complexity of the tumoral masses *in vivo*. However, to study cancer 3D models’ dynamics new technological approaches are required. Most of the deaths related to cancer are caused by metastasis but still many of the metastatic driving processes remain unknown. A fundamental player in the metastatic process is the cytoskeleton. The polymerization of actin monomers in filaments, known as F-actin, is crucial for cell motility. Also, it can be used to detect necrosis, since F-actin is exposed on necrotic cells due to the loss of the cell membrane’s integrity. To date, studies of actin dynamics in cancer cells have primarily relied on simplistic 2D models and fluorescence microscopy.

**Methods:**

In this paper, we propose combining light sheet fluorescence microscopy (LSFM) with colorectal cancer (CRC) and non-small cell lung carcinoma (NSCLC) spheroids to study F-actin distribution and exposition with minimal distortions.

**Results:**

We identified 6 different areas of F-actin intensity that could be correlated with the proliferative, senescence and necrotic zones previously described in cancer spheroid models *in vitro*.

**Conclusions:**

Our findings proved the power of the proposed LS meso aspheric optics approach to visualize and quantify F-actin in 3D cancer models with a high level of detail. Importantly, our findings also facilitate the assessment of the necrotic area's extent, clearing the path for improved anti-metastatic treatments and more accurate patient prognosis evaluation.

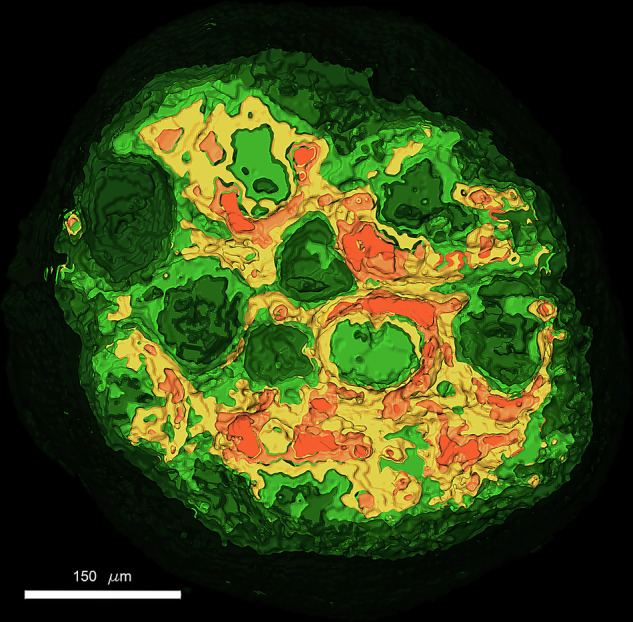

## Introduction

CRC is the second most common cause of cancer related world- wide affecting both males and females. According to GLOBOCAN, colorectal cancer is the second most common type of cancer in women and the third common one in men [1]. This type of cancer develops in the colon or in the rectum with adenocarcinoma being the most common subtype [2]. When the colorectal cancer cells proliferate and metastasize to other tissues the patient’s chances of survival are drastically reduced. Some patients with CRC present synchronously with lung cancer and vice versa. Although the number of cases are sparse [3, 4]. Besides, the most common places for CRC metastasis are liver (50%) and lung (20%). An important process related to metastasis is the epithelial mesenchymal transition (EMT). The EMT involves the activation of multiple genes and entitles severe changes in the cancer cell morphology, which facilitate the acquisition of migratorily properties in the tumoral cells [5]. It is well known that different cytoskeleton proteins interact during the metastatic process. In this dynamic process, actin filaments play a crucial role [6]. The metastatic process in solid tumors involves the dissemination of cells from the primary tumors to the lymphatic and circulatory system. Once in circulation, the cells travel and colonize other tissues in the body. To move from solid tissues into the circulatory system and seed metastatic masses in distal tissues, the activity of the cell cytoskeleton plays a preponderant role. The formation of F-actin is directly related with the motility capacities of cells in health and disease [7]. The accumulation of F-actin in the direction of the cell migration has been shown in 2D cultures [8]. Additionally, the formation of structures such as filopodia and F-actin arc-like structures, known as lamellipodia has been reported [9, 10]. However, these 2D models are overly rigid and basic. Besides, they differ significantly from the 3D structure of the tissues in the human body and do not accurately reflect the physiological reality. It is also known that the F-actin is exposed on necrotic cells due to the loss of the cell membrane’s integrity [11]. The necrosis appears in the center of the tumors, because of an intense proliferation rate of the cancer cells. The cell’s access to the nutrients and oxygen resources becomes limited in the center of the tumor, which leads to the death of these cells through necrosis. The molecules released by the necrotic cells result in the formation of new vessels and a high-rate proliferation of the rest of the still alive cancer cells. To detect the F-actin exposition in the necrotic cells it is of great relevance, since the development of a necrotic core in primary tumors is associated with a poor prognosis of the patient´s [12, 13]. Recently tumor necrosis at the tumoral masses core was associated with cancer dissemination [14].

Tumoral models like spheroids and organoids also present the formation of a necrotic core [15], which make them an interesting approach for more precise understanding of cytoskeleton dynamics and necrosis core formation in tumoral masses. Additionally, it may facilitate new paths for the development of targeted drugs to prevent metastasis in solid tumors [16]. Furthermore, as a proof of concept, to establish novel methodologies for the detection of necrosis in primary tumors.

### 2D vs 3D models in cancer research

To test new drugs and study how cancer originates and evolves in vitro, both 2D and 3D models are used [17]. In 2D approaches, cells grow in a monolayer on the top of plastic or extracellular matrix proteins, which are covered by the cultivation medium. This static environment is a highly simplified model and fails to recapitulate the complexity of solid tumor morphology and composition present in the human body. Lately, 3D tumoral models, including spheroids and organoids, have gained traction in biomedical research [18]. These models mimic the three-dimensional structure of tumoral masses and exhibit greater complexity than 2D cultures by forming multiple layers. Cells in the 3D models interact in a way that more closely resembles their behavior in the human body. When combined with microfluidic systems (cancer on chip) [19], 3D tumoral models can provide a powerful tool for studying the tumoral biology *in vitro*.

In brief, 3D cancer modelling is an exciting area of research. However, using 3D models is a challenging task, as most experimental techniques were designed for 2D cultures. For instance, studying cytoskeletal dynamics and the formation of the necrotic cores during the metastatic process via microscopy can be challenging in 3D models. Before imaging, the samples need to be fixed and embedded, a procedure that preserves the tissue structure and allows it to be sectioned into slides of 4-5 micrometers thickness. The tissue slides are then mounted onto object slides and stained prior to imaging [20]. This 2D imaging approach is complex and limits the ability to effectively study the three-dimensional morphology of the tissue samples. Although it is principally possible to reconstruct volumetric data from 2D images using computational approaches (e.g. the Whole Slide Imaging (WSI) software), this method is prone to generating artifacts and loss of information [21].

### Light sheet microscopy

A state-of-the-art technology that allows for the evaluation of 3D tissues without the mentioned shortcomings is light-sheet fluorescence microscopy (LSFM). In LSFM a thin sheet of laser light illuminates the specimen perpendicularly to the observation path of the microscope, restricting fluorescence excitation to a thin layer within the specimen, while layers below and above remain in the dark. By separating the illumination and observation pathways, a pronounced optical sectioning effect is achieved [22]. In most variations of LSFM, the laser light is initially guided through a magnifier, or an expansion unit formed by two or three optical lenses. The outgoing expanded beam then passes a second optical unit comprising a cylindrical lens and an objective. The objective focuses the light in one direction without altering it in the perpendicular direction, resulting in a thin sheet of light [23]. The more tightly the light sheet is compressed at the focal point of a conventional LSFM system, the more quickly it diverges beyond the focal point. This yields a light sheet with short Rayleigh range and generally poor uniformity along all axes [23–26].

A narrow-slit aperture can be used to increase the Rayleigh range, but this inevitably increases the thickness of the light sheet and reduces its intensity [23, 27, 28].

To overcome the drawbacks of a standard LSFM system, which typically consists of a single cylindrical lens and a slit aperture, we utilize meso-aspheric optics to generate a distortion-free image with minimal aberrations. Meso-aspheric elements, such as symmetrical conic lenses, conic-aspheric prisms, or Powell lenses, efficiently reshape the incident laser beam with classical Gaussian intensity distribution into a donut-shaped or bone-shaped beam with a semi-uniform intensity distribution [29–33].

Through the utilization of meso-aspheric elements in conjunction with other lenses, it is possible to generate an exceptionally thin light sheet characterized by an extended Rayleigh range and high uniformity, all with minimal energy loss. This is facilitated by a system that does not require a hard-edge aperture.

## Material and methods

### Spheroids generation

For the generation of the CRC and NSCLC spheroids, we cultivated Caco-2 (gender male), HT-29 (gender female) and NCI-H460 (gender male) cell lines (ATCC) in NunclonTM SpheraTM 96-well plates (Thermo Fisher Scientific Inc). We seeded the cells at doses 20.000 cells per well, and cultivated them in an atmosphere containing 5% CO_2_ at 37 °C. Next, we induced the formation of the spheroids with a basal medium consisting of Dulbecco’s Modified Eagle’s Medium/F12 and Ham’s media (Sigma-Aldrich) in a proportion 1:1 with 3.151 g/L glucose, L-Glutamine, 10% v/v bovine serum EU standard and Pen Strep (Sigma-Aldrich), at a working concentration of 100 units of potassium penicillin and 100 μg of streptomycin sulfate per 1 mL of culture medium. The medium was supplemented with 5 μg/mL insulin (GibcoTM), 10 ng/mL Fibroblast Growth Factor-Basic (bFGF, Merck KGaA) and 20 ng/mL of Epidermal Growth Factor (EGF, Sigma-Aldrich). The medium used for the spheroid formation was changed every two days. We cultivated the spheroids until day 7. Then, they were stained and prepared for microscopy evaluation.

### Spheroids viability assays

Caco-2, HT-29 and NCI-H460 spheroids were generated from early passages in 96 well anti-adherent plates as we described previously. The viability was evaluated after 24, 48, 72 hours, and 7 days in culture by using the CCK8 kit from (MedChemExpress LLC, Austria) and the Enzo Absorbance 96 Plate Reader (Enzo Life Sciences, Inc., USA). We added the CCK8 solution in a ratio 1:10 to the samples and incubated them for 2 h. After that we transferred the supernatant to a clean plate and proceeded with measuring the light absorbance at 450 nm. Next, we calculated the cell viability using the following formula:

$${{\rm{Cell}}}\, {{\rm{viability}}}\, (\%)=({{\rm{Treatment}}}\, {{\rm{signal}}} / {{\rm{Control}}}\, {{\rm{signal}}}) \times 100$$

Each experiment was replicated 3 times. For the statistical analysis we used the software GraphPad Prism 9. version 9.4.0. We did a One-Way ANOVA (alpha = 0.05) ****P Value < 0.0001, ***P Value 0.0007 and ns. *P* Value 0.3158.

### Spheroids preparation for microscopy

Spheroids cytoskeleton staining. To enable the evaluation of the cytoskeleton in CRC, and lung cancer spheroids, we fixed the generated spheroids with 4% paraformaldehyde (Sigma-Aldrich) and permeabilized them with 0.5% Triton X-100 (Merck). Subsequently, we stained the cell’s cytoskeleton with phalloidin-488 (Abcam), which binds to F-Actin in the cells.

Spheroids inclusion in agarose. After staining, we embedded the spheroids in 3% transparent low melting agarose (Thermo Fisher) and let the sample solidify overnight at 4 °C.

Spheroids preparations assembly. The following day, we removed the agarose blocks containing the spheroids from the molds and attached them to small metal blocks for securing them during the imaging procedure. The metal pieces were glued to the samples using two-part epoxy glue. The glue was allowed to cure at 4 °C in a Petri dish for 12 h. Finally, the samples were imaged using our meso-aspheric light-sheet microscopy system and a NIKON ECLIPSE TE200 microscope equipped with a Hamamatsu camera system.

Media absorbance evaluation. Prior to imaging with our meso-aspheric light sheet microscope, the samples were immersed in a glass chamber filled with a solution with a refractive index closely matching that of the specimen. To determine the optimal immersion medium, we measured the light absorbance of the samples in three different media: M1 (CRC basal medium), M2 (Fibroblast medium) and M3 (Phosphate buffered saline, PBS). This evaluation was done at light wavelengths of 405, 488, 532 and 670 nm using a plate reader. Eight samples were evaluated for each solution. Our results indicated that the M3 (PBS) solution was the most suitable solution for image acquisition.

### Light sheet microscopy setup

In general, the distribution of a laser beam, which can be approximated by the fundamental Gaussian mode, at any arbitrary point along the propagation axis is described by Eq. (1):

1 $$\psi ({r,z}) 	 = {\psi}_{0}\frac{{w}_{0}}{w(z)} {e}^{\frac{-{r}^{2}}{{w(z)}^{2}}} {e}^{i({kz}- \eta ({z})+\frac{\pi {r}^{2}}{\lambda R({z})})} \\ 	 = {\psi }_{0}{{e}^{\left(\frac{-{r}^{2}}{{w}_{0}^{2}+{\left(\frac{\lambda z}{\pi {w}_{0}}\right)}^{2}}\right)}}e^{i\left(\frac{2\pi z}{\lambda }-\left(\frac{z\lambda }{\pi {w}_{0}^{2}}\right)\right)}{e}^{i\left(\frac{\frac{\lambda z}{\pi }{r}^{2}}{{w}_{0}^{4}+{\left(\frac{\lambda z}{\pi }\right)}^{2}}\right)}$$

where *w*_*0*_, *λ*, and *z* are the beam waist, wavelength, and direction of propagation, respectively.

Additionally, key beam parameters such as phase ($\eta (z)$), wavefront radius curvature (R(z)), and amplitude of the transverse profile, are given as $\eta \left(z\right)=\arctan (\frac{z \lambda }{\pi {w}_{0}^{2}})$, $R\left(z\right)=z(1+\frac{{\pi }^{2}{w}_{0}^{4}}{{\lambda }^{2}{z}^{2}})$, and ${e}^{\left(\frac{-{r}^{2}}{{w}_{0}^{2}+{(\frac{\lambda z}{\pi {w}_{0}})}^{2}}\right)}$, respectively [29, 34].

By changing the phase, amplitude, and wavefront curvatures we can reshape the light distribution profile of the incident laser beam [32, 35–42].

**Fig. 1:**
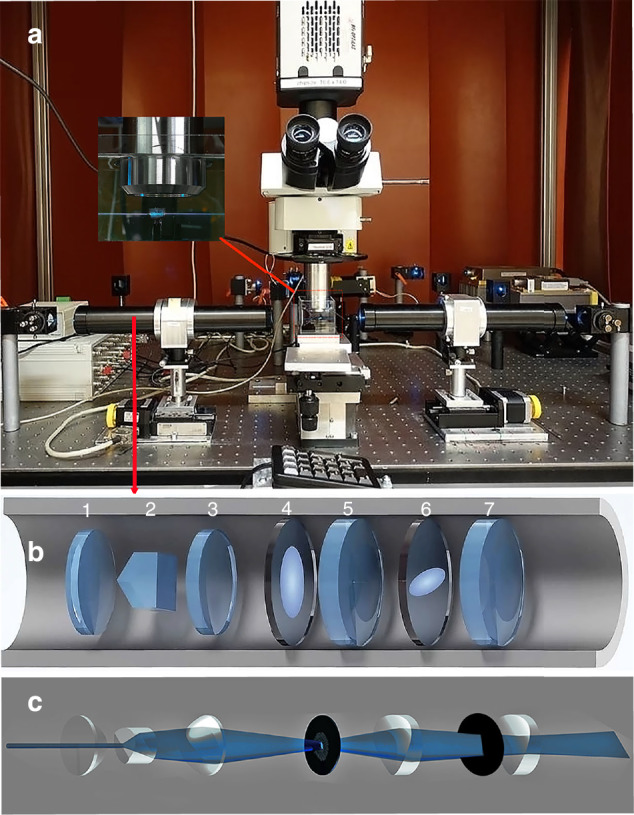
Microscopy setup. **a** Simplified Meso-aspheric light sheet microscope. **b** The technical drawing of the Light sheet generator unit-LSGU comprising: 1) Aspheric condenser lens with an 18 mm focal length (Qioptiq, Germany). 2) Conic aspheric cylindrical prism with vertical plane of symmetry having a fan angle of 7.5° known as line generator lens (Laserline, Canada). 3) Further aspheric condenser lens with 18 mm focal length (Qioptiq, Germany). 4) A symmetrical soft aperture designed to remove undesired scattered light distributed far away from the optical axis. 5) An achromatic cylindrical lens of + 80 mm focal length (Qioptiq, Germany). 6) A second soft aperture, featuring an elliptical shape, is employed to achieve a gradual attenuation of light distribution in an elliptical manner. This enhances the quality of the light by effectively eliminating undesired scattered light distributed far away from the center of the optical axis. 7) Another achromatic cylindrical lens of + 80 mm focal length (Qioptiq, Germany). **c** Alteration of the incident laser beam from Gaussian distribution into a thin light sheet.

Our light-sheet based imaging system as shown in Fig.1a is composed of several key components: the laser light source, the light-sheet generator unit (LSGU) featuring both aspheric and Axial meso-aspheric optical elements meticulously arranged on a computer-controlled horizontal positioning system. The vertical positioning unit is responsible for moving the specimen container along the detection axis, which is perpendicular to the LSGU. The imaging system encompasses a corrected objective for refractive index mismatch, a tube lens, a camera, and a computer.

The illumination source is a Sapphire laser emitting a 488 nm beam with Gaussian intensity distribution with output beam width of 0.7 mm (Coherent Inc., Germany). The beam divergence is less than 1.2 mrad and the beam propagation factor (M^2^) is approximately 1.2, while the power can be adjusted up to 500 mW. Using a 50% beam splitter the laser beam is divided into two beams of almost equal intensity.

These beams are guided through two identical optimized LSGUs as shown in Fig. 1b and reshaped to a very thin sheet of light as shown in Fig. 1c.

The optimized LSGU comprises an aspheric condenser lens that focuses the beam on the axial conic aspheric line generator to reshape the laser beam to a bone-shaped profile with semi-uniform distribution. However, the fan angle of the axial conic aspheric lens directly influences the quality of this semi-uniform bone-shaped beam. Authors demonstrated that among a wide range of tested axial-meso-aspheric lenses, our results confirmed that lenses with fan angles of 10° and 7.5° when integrated with additional aspheric components, exhibit well-structured light sheet that could be utilized for imaging of biological samples of various sizes in light sheet fluorescence microscopy technique [34]. However, a 7.5° axial-meso-aspheric lens exhibits less fluctuations and more uniformity in the beam intensity distribution compared to a 10° lens [34]. The conic tip of the second element (7.5° axial-meso-aspheric lens) is placed at ~17.5 mm distance from the flat surface of the first lens. Placing another lens identical to the first element at ~a 36 mm distance enables us to obtain a beam with an elliptical intensity distribution. To optimize this elliptical beam a custom-made soft aperture is placed between the third and fifth elements which is placed at a 98 mm distance from the flat back surface of the third optical element. This soft aperture has customizable density gradients that are formed elliptically from the center to the outside of the component stretching along the y-axis. The cylindrical lens compressed the beam width along one axis while having almost no distinguishable effects on the perpendicular direction. For further improvements, the beam is guided toward another soft aperture that is located between the first cylindrical lens and another identical cylindrical lens that is placed at ~113 mm distance from the flat surface of the first cylindrical lens. The filter also has customizable density gradients that are formed elliptically from the center to the outside of the component perpendicular to the direction of the first soft aperture, item 4. Both filters are used to eliminate undesired intensity variations in the optical system and to modify the wavefront of the optical source [23–25]. The last cylindrical lens generates a thin stable sheet of light at its focal distance. Both LSGUs are placed on a computer-controlled linear stage (LS-65, PI-Micos, Germany) at the opposing sides of the specimen chamber made of quartz. The specimen chamber is located on a computer-controlled vertical elevation stage with an adjustment precision of 100 nm (Es-100, PI-Micos GmbH, Germany) and a manual adjustable cross table for adjusting the position of the sample in x and y-direction. The specimen chamber containing the sample is filled with a clearing solution matching the refractive index of the specimen [29].

The light-detection system for imaging includes a customized microscope with modified objectives, a computer-controlled filter wheel with different optical band-pass filters, and a scientific-grade CCD Camera (Andor-Neo, Andor Technology, Ireland).

Image recording is controlled by custom-made software allowing an automated recording of image stacks. Once the stack of images has been acquired, deconvolution is performed using the software NeuroDeblur (MBF Bioscience, USA) followed by 3D reconstruction with Amira 5.2 (ThermoFisher, USA).

### Evaluation of the formation of the light sheet

#### Comparative visualization analysis

To assess the quality of the light sheet generated by the simplified Meso-aspheric Light Sheet Microscope, a comparative visualization analysis with a standard system is conducted. In this evaluation, a glass chamber (5 cm x 5 cm x 5 cm, Helma Optics, Germany) is filled with water, and one drop of Fluorescein sodium salt, a fluorescent label and water-soluble form of fluorescein is added to facilitate the comparison between the two systems.

The output beam from the Sapphire laser (Coherent Inc./Germany, 500 mW, 488 nm) is split into two equal portions using a 50% beam splitter. Subsequently, these divided beams are directed towards two tubes positioned on opposite sides of the specimen chamber, facing each other.

**Fig. 2:**
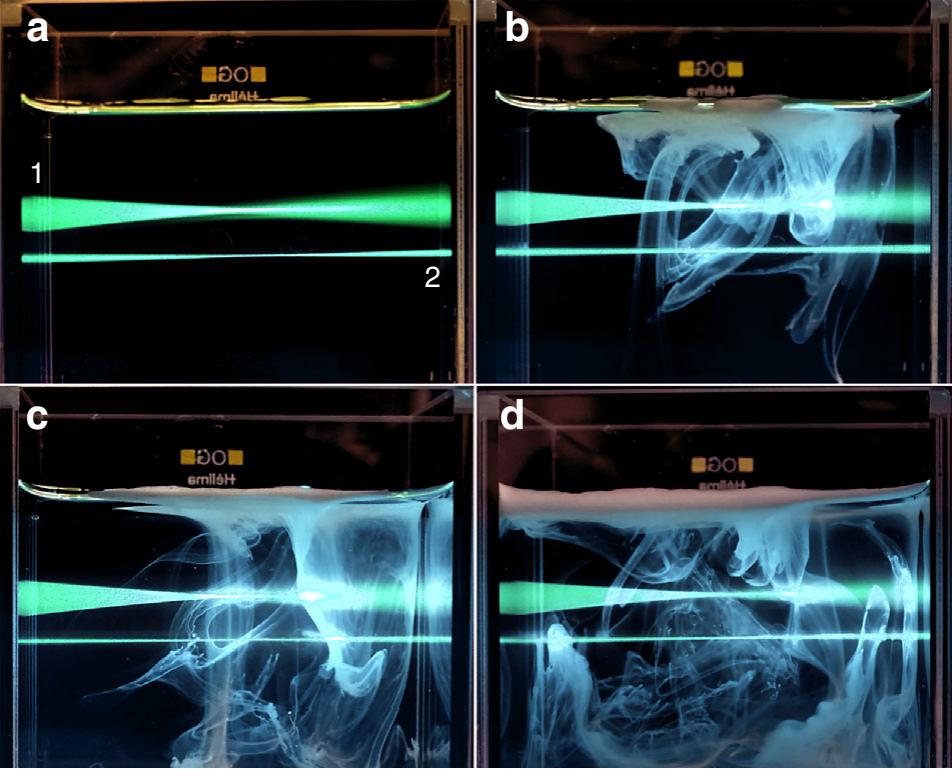
Comparative visualization. Visual comparison of two light sheets, where (1) corresponds to the standard system, and (2) represents the light sheet formed by the meso-aspheric-based light sheet microscope. **a** Illustrating a side-view of a glass chamber containing 110 ml water and a drop of fluorescein sodium, simultaneously illuminated by the standard light sheet (from the left side) and the meso-aspheric-based light sheet (from the right side) when the medium is transparent, **b**–**d** Recording the effects of introducing one drop of milk (3.5% fat) into the medium on both light sheets over a 60-second time frame with 20-second intervals.

One tube represents the standard system, consisting of lenses that generate a parallel magnified beam. This beam is directed onto an 8 mm × 25 mm rectangular aperture, followed by a 60 mm achromatic cylindrical lens. The combination ensures the production of a light sheet characterized by a well degree of uniformity, minimal energy loss, and limited hard edge aperture effects. This light sheet illuminates the chamber from the left side, identified as number 1 in Fig. 2a.

On the opposite side, the second tube incorporates an axial meso-aspheric element in conjunction with aspheric lenses, achromatic lenses and soft apertures, as illustrated in Fig. 1b. This arrangement illuminates the chamber from the right side, identified as number 2 in Fig. 2a.

Considering that most biological samples are not fully transparent, it becomes crucial to conduct visual analyses in mediums with varying degrees of turbidity. To demonstrate the influence of turbidity on the light sheet at the focal point and along the propagation axis, a drop of milk (3.5% fat) is introduced into the medium. Side-view effects are documented at 30 s intervals, as illustrated in Fig. 2b–d. The light sheet generated by an axial meso-aspheric based system not only demonstrates superior optical characteristics and quality in a clear medium compared to the standard system but also maintains its high quality even when in contact with a turbid medium.

#### Construction of 2D and 3D images using Amira Software

With Thermo Scientific Amira-Avizo Software (2020.2), we can process stacks of captured images to generate high-resolution 3D visualizations and perform in-depth analyses of complex biological data. This software allows us to uncover intricate details of both internal and external tissue structures, providing valuable insights into their composition and organization.

By capturing a stack of images at defined intervals along the detection axis using an optimized objective (e.g., 4X XLFLUOR4X/340, NA: 0.28, Olympus/Japan) equipped with a modulator to compensate for refractive index mismatches between air, the medium, and chemically cleared samples, we can reconstruct the dataset in Amira-Avizo to reveal fine structural details of the sample’s outer and inner regions (e.g., Fig. 1a–b, Supplementary A).

#### Segmentation analysis

The emitted signals detected by a high-dynamic-range CCD sensor (e.g., Andor-Zyla, Andor Neo) after excitation can be used for quantitative analysis. The captured image reveals intensity variations based on autofluorescence from different materials, reflecting differences in biochemical composition. This enables segmentation of regions with distinct energy levels. We begin by applying thresholding to separate bright and dark regions, followed by clustering and grouping pixels with similar fluorescence intensity. Additionally, spectral analysis is performed to classify materials based on their autofluorescence emission spectra.

## Results

### Spheroids viability assays

**Fig. 3:**
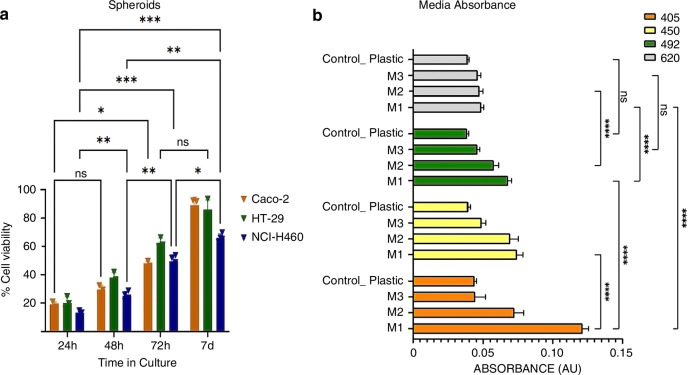
Spheroids viability. **a** *Cell viability during Caco-2, HT-29 and* NCI-H460 spheroid formation. The viability was evaluated with the CCK8 metabolic assay after 24, 48, 72 h, and 7 days in culture. *N* = 3 samples. For statistical analysis we did a One-Way ANOVA using GraphPad Prism 9.4.0 The chosen level of significance was α = 0.05 ****P Value < 0.0001, ***P Value 0.0005 and ns *P* Value 0.1230. **b** Plastic and Media light absorbance at 405, 450,492 and 620 nm. Three different media were evaluated: M1 = CRC basal medium, M2= Fibroblast medium and M3 = PBS. The absorbance of the empty cuvette filled with air was accessed separately and subtracted from the measurements. For statistical analysis a Two-Way ANOVA (alpha = 0.05) was done using GraphPad Prism version 9.4.0. ****P Value < 0.0001

Before staining and embedding the spheroids in agarose we evaluated the viability of our 3D tumoral models after 24, 48, 72 h and 7 days in culture, as described in Materials and Methods. Our results in Fig. 3 show that the different types of spheroids were proliferative and viable after 7 days in culture with viabilities of 90% (Caco-2), 86% (HT-29) and 70% (NCI-H460). Besides they exhibited the characteristic compact and spherical shape Fig. 3a

### Sample mounting process for imaging

After the viability evaluation we fixed and stained the cytoskeletal F-actin of the CRC spheroid cells as described in Material and Methods. Afterwards, we embedded the spheroids in transparent agarose and glued the samples on a piece of sheet metal for fixating them during imaging, Fig. 1a–d (Supplementary).

### Media absorbance evaluation

For imaging the mounted samples were submerged in a glass cuvette filled with the imaging medium. To find the medium that best possibly matches the refractive index of the specimen, we measured the light absorbance of the immersed samples in three different media: M1 (CRC basal medium), M2 (Fibroblast medium) and M3 (Phosphate buffered saline, PBS) at wavelengths of 405, 488, 532 and 670 nm as described in the Material and Methods section. The results are shown in Fig. 3b. Based on the obtained results Phosphate buffer saline (M3) was chosen for imaging.

### Enhancing light sheet evaluation by specimen visualization

To further evaluate our findings, we captured images depicting the cytoskeleton F-actin in CRC (Caco-2, HT-29) and lung cancer (NCI-H460) spheroids.

**Fig. 4:**
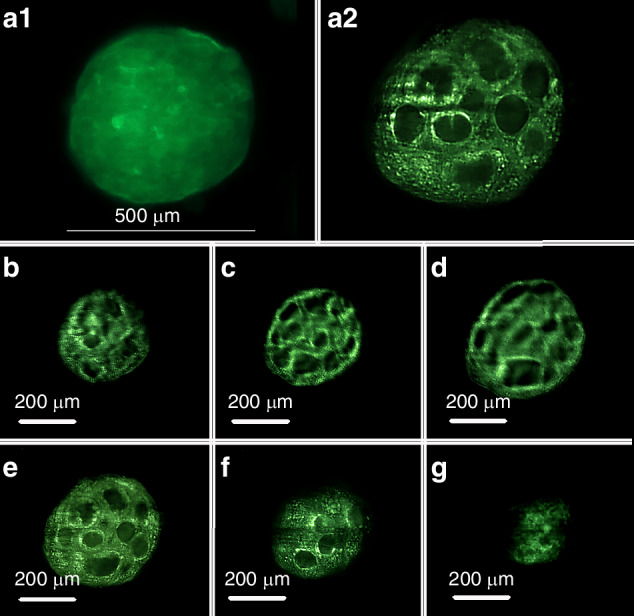
Images of the cytoskeleton F-actin of CRC spheroids. **a**1 Image of Caco-2 spheroids embedded in agarose, acquired using a NIKON ECLIPSE TE200 microscope equipped with a Hamamatsu camera. A2) Image obtained using the optimized one-axial Meso-Aspheric-based light-sheet microscope, reconstructed from a stack of 70 images captured with an Andor Neo camera at 2 µm intervals. **b**–**g** Six images captured along a 90 µm thickness with 15 µm intervals along the detection axis, starting 50 µm from the first recorded image of the deepest plane, revealing intricate inner structural details.

Initially, a 2D image of Caco-2 spheroids embedded in agarose was acquired using a NIKON ECLIPSE TE200 microscope equipped with a Hamamatsu camera (Fig. 4a1). In the next phase, we employed our optimized light-sheet microscope with a 4X objective (XLFLUOR4X/340, NA: 0.28, Olympus/Japan) NA: 0.28, Olympus/Japan), incorporating a modulator to compensate for the refractive index mismatch between air and the immersing medium. The sample was illuminated from both sides by two thin light sheets.

A computer-controlled horizontal stage was used to position the light sheet at its minimum width on the sample, while a vertical stage scanned the sample through the light sheet, capturing 70 images at 2 µm intervals. The resulting stacked image (Fig. 4a2) reveals intricate surface details Fig. 2a–B (Supplementary). Notably, the light-sheet fluorescence microscope excels in visualizing fine internal structures, as demonstrated in Fig. 6a2–g. For the sake of initial visualization of autofluorescence, a pure simple green color map is used.

**Fig. 5:**
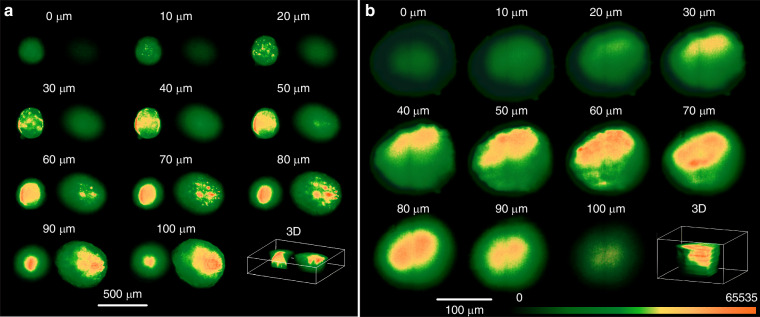
Autofluorescence distribution across different regions. XY intensity distribution of spheroids over 100 µm depth according to the colormap between 0 to 65535 levels: **a** Two neighboring CRC spheroids HT-29. **b** A lung cancer spheroid NCI-H460.

To differentiate the distribution of autofluorescence across different regions in 2D and 3D, we applied a colormap to enhance visualization of autofluorescence patterns in various samples, including CRC and lung cancer. Using a 10X objective (NA: 0.3, Olympus/Japan), we captured 200 images with 0.5 µm interval over a depth of 100 µm along the detection axis, as shown in Fig. 5a, b.

Mov1 and Mov2 demonstrate 3D distribution of the autofluorescence across the samples shown in Fig.5 according to the new colormap.

### Surface occupied by F-actin in the CRC spheroids

**Fig. 6:**
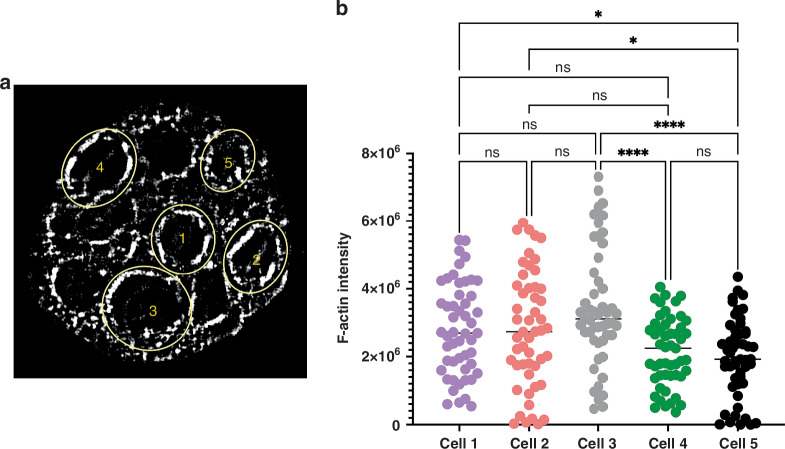
F-actin distribution and quantification in 5 cells in CRC spheroids. **a** example image of the F-actin presence and distribution in CRC spheroids. The F-actin is highlighted in white. **b** Quantification of the F-actin in our images. *N* = 51 images per cell. For statistical analysis we did a One-Way ANOVA using GraphPad Prism 9.4.0 The chosen level of significance was α = 0.05. * *P* Value 0.0300, ****P Value < 0.0001.

The density and distribution of the F-actin can be linked with the migration and invasive capacities of the cancer cells in CRC [7]. To evaluate that in our 3D spheroids we used the Thermo Scientific Amira-Avizo Software (2020.2) as described in the Material and Methods section. In Fig. 6a we showed the distribution of the F-actin in 5 different cells. Also, in the Images 0 to 50 Supplementary we show the F-actin distribution in a stack of 51 images. Besides, Mov3 supplementary, created from the recorded images obtained by the meso-aspheric based light sheet microscope, vividly depict the F-actin structural details and distribution throughout the sample. Our results show that in the CRC spheroid cells the F-actin is distributed around the edge of the cell membrane in fragments of different sizes. This kind of F-actin distribution was reported before in other types of cancer cells [43]. It is known that the EMT relates to the intracellular actin dynamics and is linked with cancer progression where cell migration is facilitated by the polymerization and polymerization of actin. Actin is present in the cells in two forms G-actin (free monomer) and F-actin (microfilament). The F-actin microfilaments can form more complex structures like bundles and branched networks. The actin bundles facilitate the cell movement in response to environmental and cellular stimuli [44]. It was reported that there is a correlation between cell stiffness and cell migratory capacities in cancer cells. Stiff cells present great motility capacities in comparison with cells with poor motility capacities that are soft. It is known that F-actin contributes to cell stiffness. For this reason, it was proposed as a biomarker for cell motility [43]. In Fig. 6b we show the quantity of F-actin in 5 cells of one of the CRC spheroids. Differences in the quantity of the F-actin are shown (Mov3) and Fig. 3*Stack of images* in (Supplementary).

### Quantitative analysis through segmentation

The signal intensity and distribution of the F-actin can be related with the exposition of the cytoskeleton in the necrotic areas of the spheroids. For quantitative analysis of the F-actin signal intensity in the 3D tumoral models, we performed 8-level segmentation using Amira software. This was applied to a stack of images of Caco-2, CRC samples, acquired with an Andor’s Neo 5.5 sCMOS camera (2560 × 2160 pixels, 16-bit depth). Each pixel in these images has an intensity value ranging from 0 to 65,535. The segmentation was designed to localize structures exhibiting autofluorescence within a specific intensity range.

To achieve this, we applied intensity thresholding based on our general colormap spanning 65,536 intensity levels. This allowed us to isolate regions in the images corresponding to different autofluorescence intensities. Following thresholding, we performed labeling and segmentation to categorize these regions into eight distinct levels, helping to identify and visualize specific biological structures based on their autofluorescence signals.

Since the spheroids are embedded in agarose, the embedding material can scatter light, generating background noise. To mitigate this, we designated the first segmentation level (0–7536) to remove the general haze caused by the agarose. The remaining intensity range was divided as follows:

Four Green Levels (low to mid-range autofluorescence):

- Green1: 7,536 – 14,786
- Green2: 14,786 – 22,036
- Green3: 22,036 – 29,286
- Green4: 29,286 – 36,536.

Three High Autofluorescence Levels:

- Yellow (Y1): 36,536 – 46,203
- Pink (P1): 46,203 – 55,870
- Orange (O1): 55,870 – 65,535

**Fig. 7:**
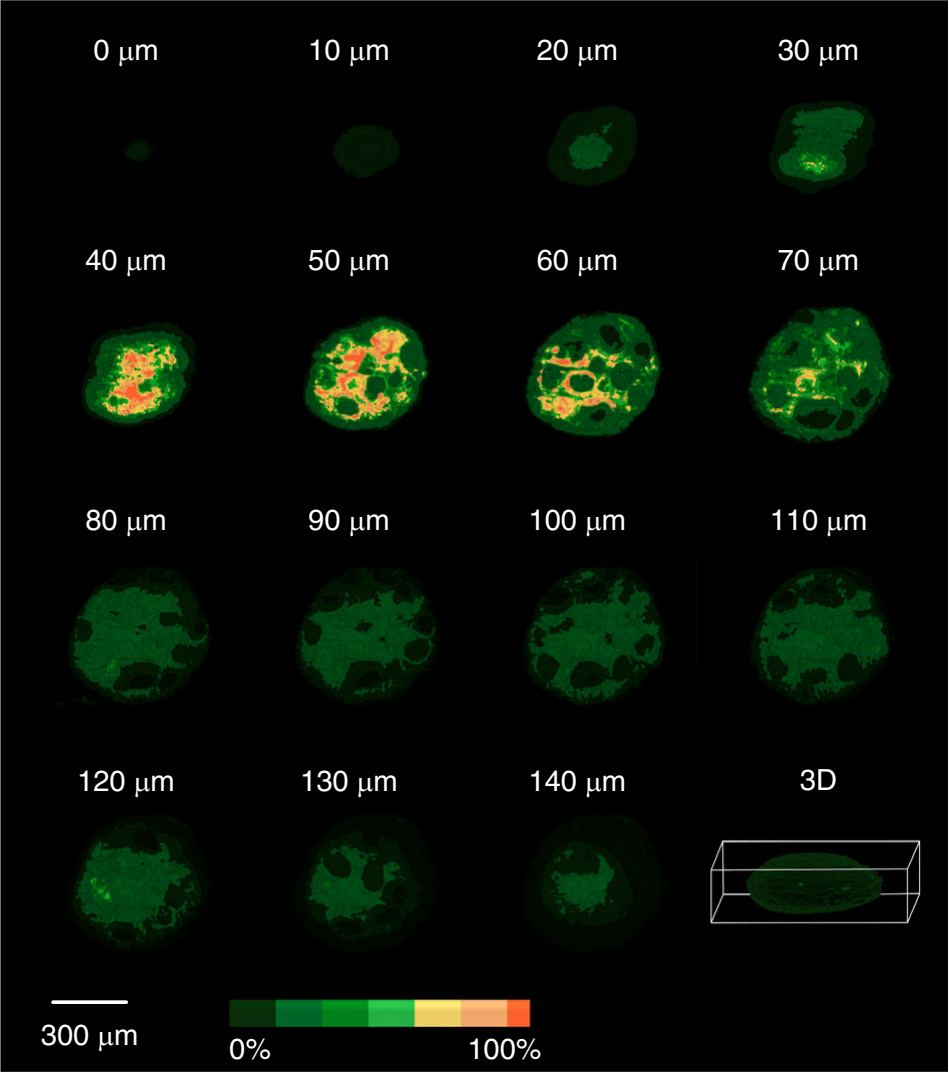
Segmented regions of a Caco-2 CRC spheroid. A stack of 70 recorded images at 2 µm intervals is used. 0 microns corresponds to the deepest part of the spheroid along the detection axis, while 140 microns represents the topmost section.

This segmentation approach enables a clear differentiation of autofluorescent structures based on intensity, facilitating the identification of biologically relevant features. Figure 7 illustrates the segmented regions of a CRC spheroid, captured from a stack of 70 images recorded at 2-micron intervals. In this dataset, 0 microns corresponds to the deepest part of the spheroid along the detection axis, while 140 microns represents the topmost section. The 3D-image of CRC-spheroid and various segmented volume along different axes is shown in Mov4.

**Fig. 8:**
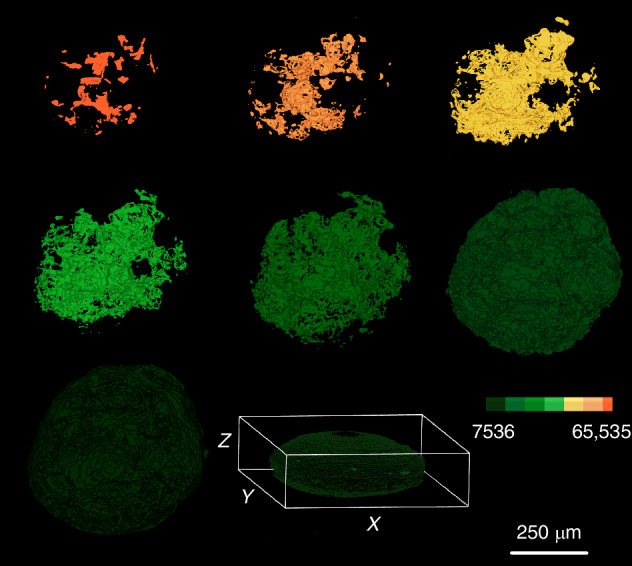
Segmentation according to emission intensity level. Volume segmentations of the sample based on intensity ranges variate by different colors.

Since the captured images are based on autofluorescence emission, segmentation is used to classify regions according to their emission intensity levels. Figure 8 displays volume segmentations of the sample based on this approach, which are represented by different colors.

Table 1 also includes a percentage-based classification, assigning numerical values to the segmented regions according to their intensity ranges. They provide a quantitative approach to characterizing autofluorescent structures based on the intensity distribution.

**Table 1:**
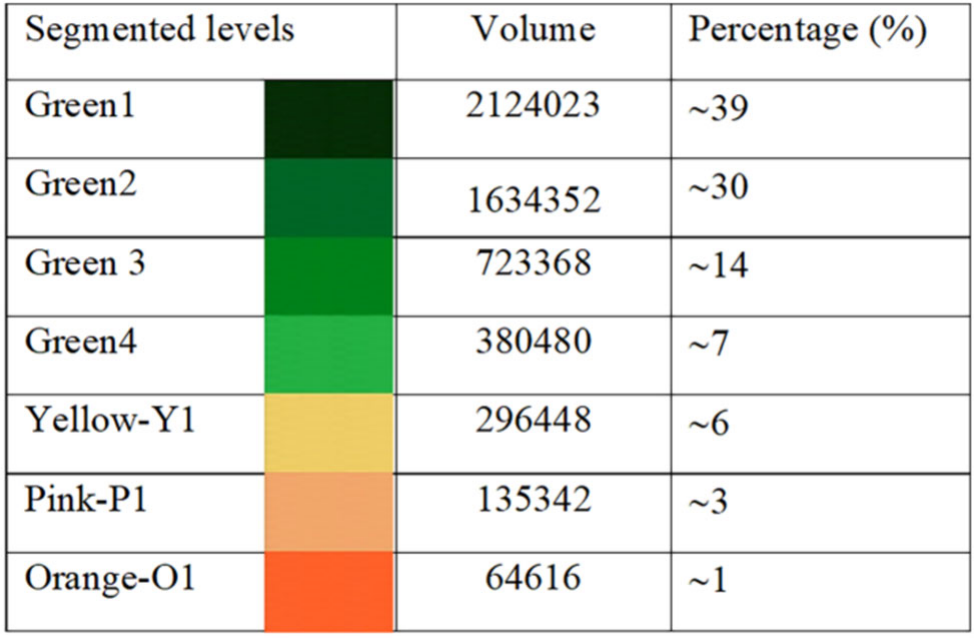
Percentage-based classification of the segmented regions by assigning numerical values to various segmented regions.

It is evident that 10% of the structure contains nearly 50% of the total intensity above the mean value.

Movie 4 was generated to observe the volume distribution of the different signal intensities from the external layer to the inner layer of the Caco-2 spheroids.

## Discussion

The use of 3D models in cancer research permits study how the disease arises and evolves in a realistic manner. However, to work with this type of model’s techniques must be adapted. Our present work proves that it is possible to investigate the F-actin cytoskeleton dynamic in CRC spheroids by using light sheet fluorescence microscopy. Also, to analyze and visualize the signal intensity distribution of F-actin stains in the spheroids multilayered structure. Our approach facilitates obtaining information layer by layer from the outside to the inner part of the spheroids. This method permits to evaluate F-actin dynamics and enriched areas without losing spatial resolution. Tumoral masses are heterogenous and contain cells with different metastatic capacities [45]. Besides, actin dynamics plays a preponderant role in the metastatic capacities of the different tumoral cells. Being involved in cell remodeling and motility capacities [8] that the cells undergo to leave the primary tumoral mass to colonize another tissue. The movement of the cancer cells is possible thanks to the polymerization and depolymerization of actin [46]. The F-actin is accumulated in the direction of the cell migration and makes the cellules stiff and more mobile [47].

The accumulation of F-actin in the direction of the cell migration has been shown very often in 2D cultures [8, 10]. However, this 2D models are too rigid and basic. Besides, they are very different from the 3D structure of the tissues in the human body. The 2D models are not aligned with reality physiologically speaking. Detailed investigations of the changes of the cytoskeleton in 3D tumoral models may help to understand cytoskeleton dynamics in a more precise manner. Importantly in the present work we showed for the first time that the intensity of the F-actin signal can be related with the presence of necrosis in CRC and lung cancer spheroids. We identified 6 different areas of intensity that could be correlated with the proliferative (Green 1, Green 2), senescence (Green 3, Yellow 1) and necrotic zones (Pink 1 and Orange 1) previously described in cancer spheroid models in vitro [48].

In the present work we prove that it is possible to study the distribution and quantity of F-actin in a more physiological related manner. Here, we showed that our LS meso aspheric optics approach has proved successful to quantify and evaluate the F-actin distribution in 3D cancer models. Importantly also to facilitate information about the extension of the necrotic area. These open new ways to study the role of F-actin in cancer and metastasis. Also, for the development of efficacious antimetastatic treatments and patient prognosis studies by evaluation of necrosis in surgical pieces.

## Supplementary information

Supplementary information

Supplementary movie 1

Supplementary movie 2

Supplementary movie 3

Supplementary movie 4

## Data Availability

No datasets were generated or analysed during the current study.
