# Supplementary material for: Cytoskeleton imaging of colorectal and lung cancer spheroids using light sheet microscopy
Source: BJC Rep. 2025 Jun 18;3:45. doi: 10.1038/s44276-025-00144-3 (PMC12177031; doi:10.1038/s44276-025-00144-3)
Supplement: Supplementary file 1 — Supplementary information [file 44276_2025_144_MOESM1_ESM.pdf]

Supplementary

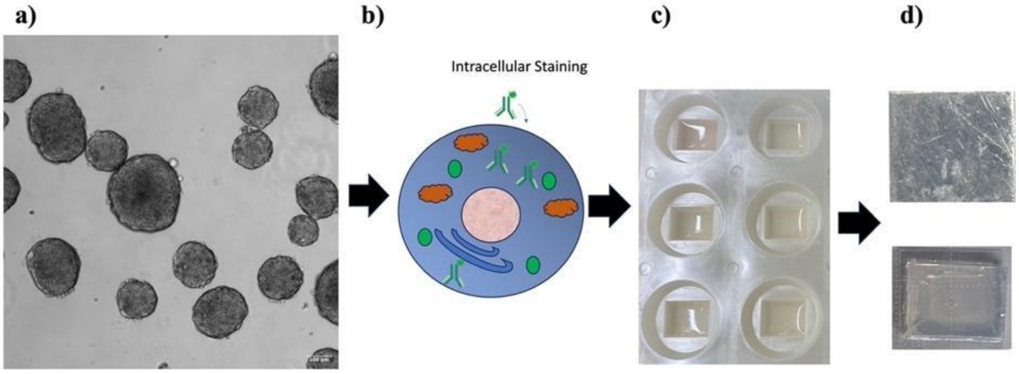

Figure 1a–d (Supplementary) . Sample mounting for imaging. a) Caco-2 spheroids (day 7) fixed with paraformaldehyde 4% (Sigma-Aldrich), permeabilized with Triton 0.5% (Merck) and b) stained with phalloidin-488 (Abcam). Phalloidin binds the F-actin in the cytoskeleton of the cells. c) After staining the spheroids were embedded in 3% transparent agarose. d)The obtained agarose blocks were glued on small metal plates and immediately imaged.

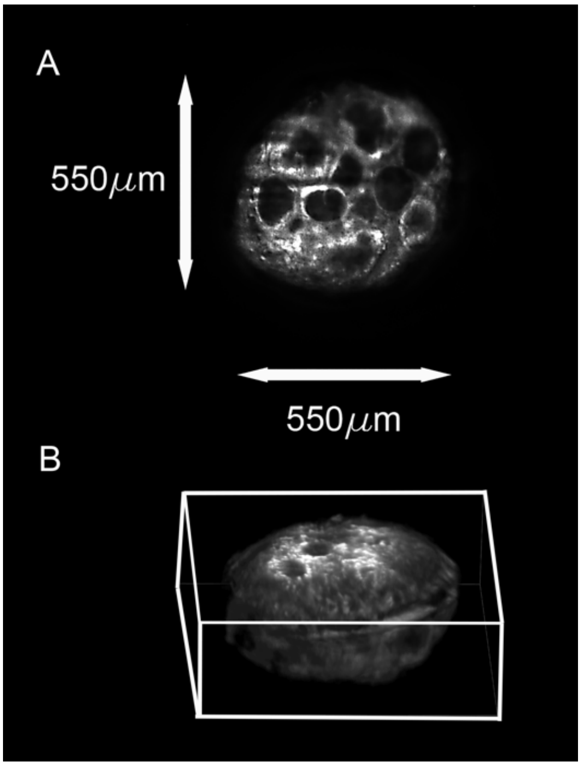

Figure 2A–B (Supplementary). A stack of 70 images was captured along the detection axis at 2 μm intervals using a 4X objective (XLFLUOR4X/340, NA: 0.28, Olympus/Japan). (A) A single 2D image along the XY plane. (B) A 3D image reconstructed from the entire stack using Amira-Avizo software. The clipping-plane mode allows navigation through the 3D image, enabling detailed visualization of each slice along the XY, XZ, and YZ planes.

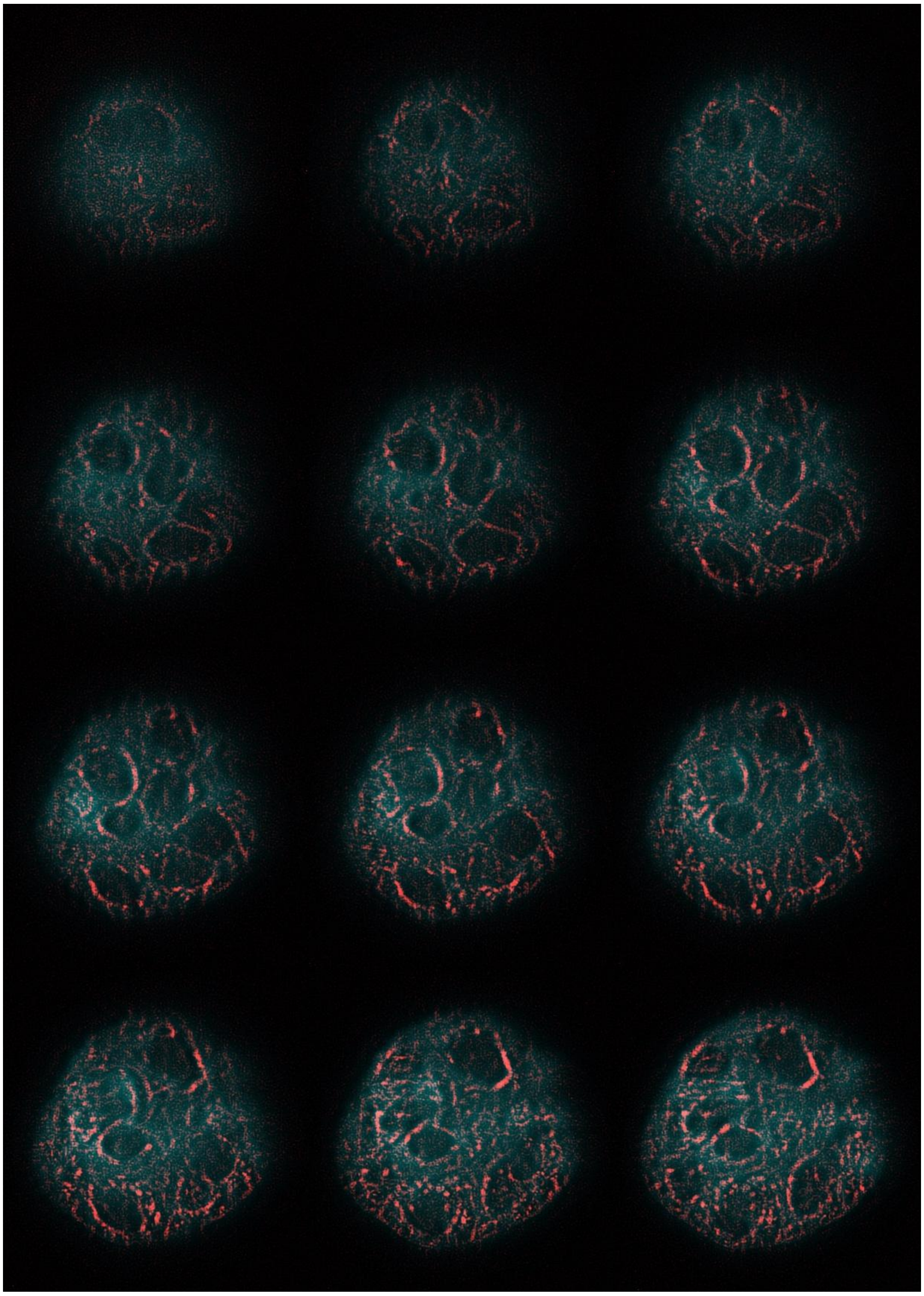

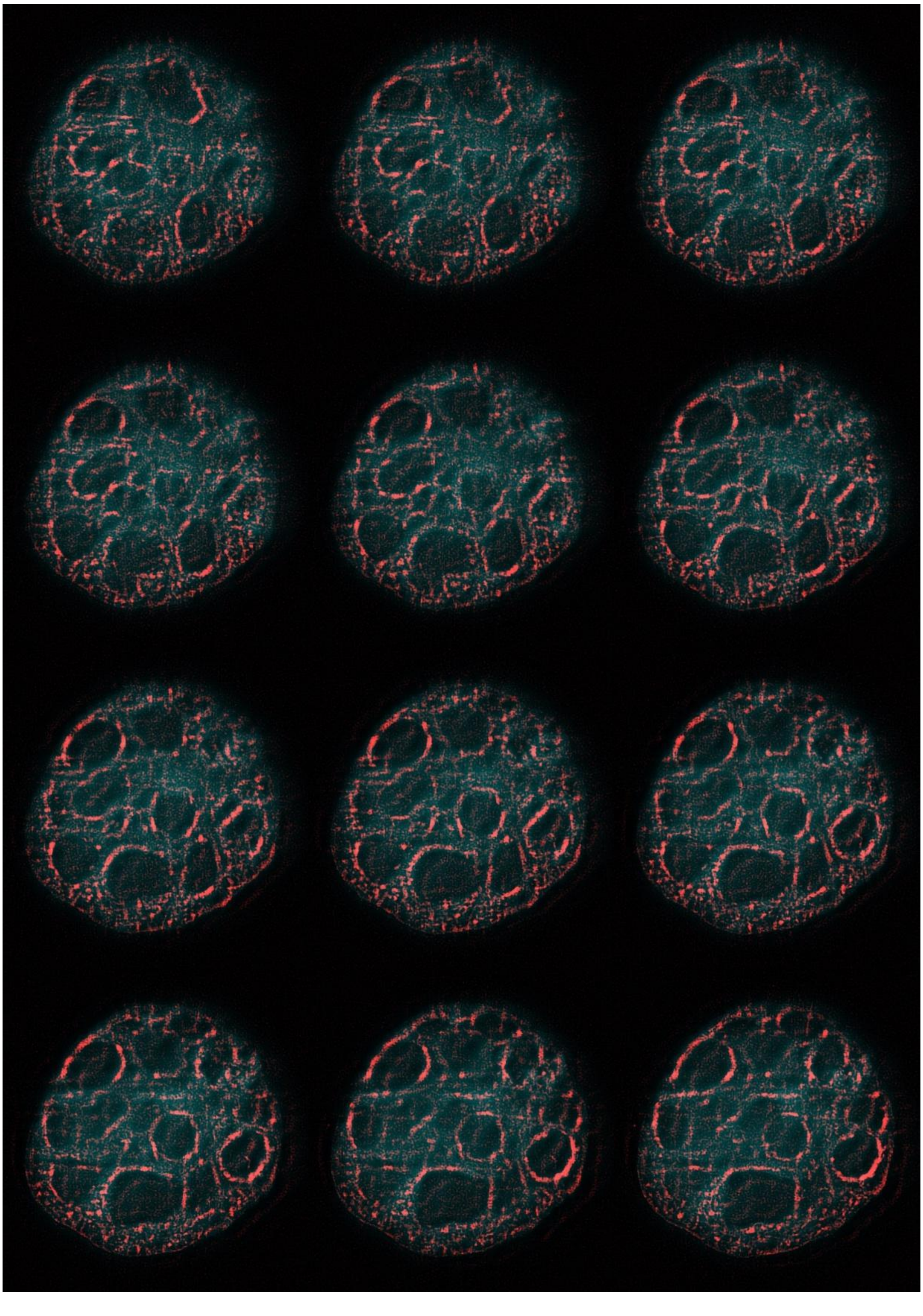

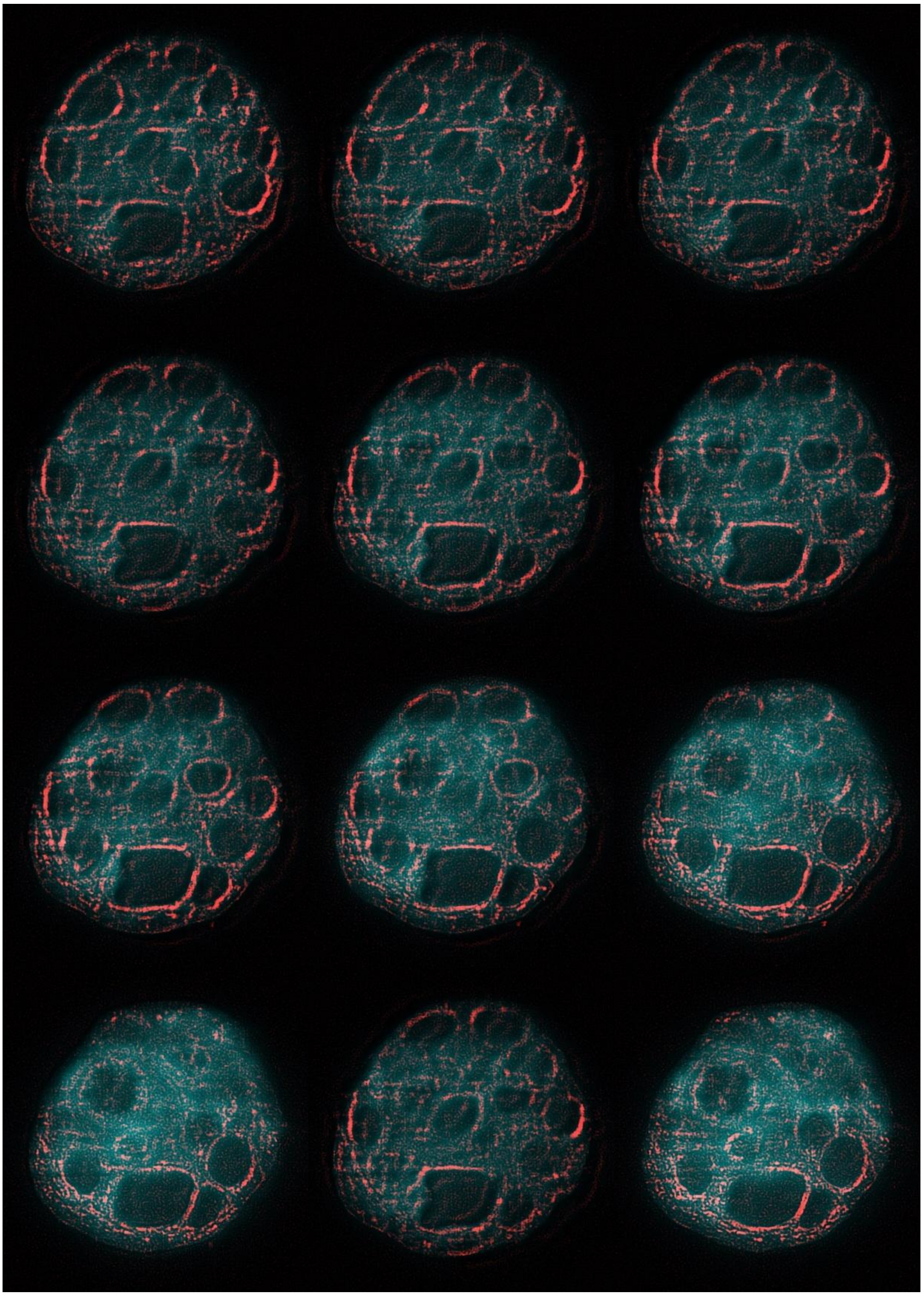

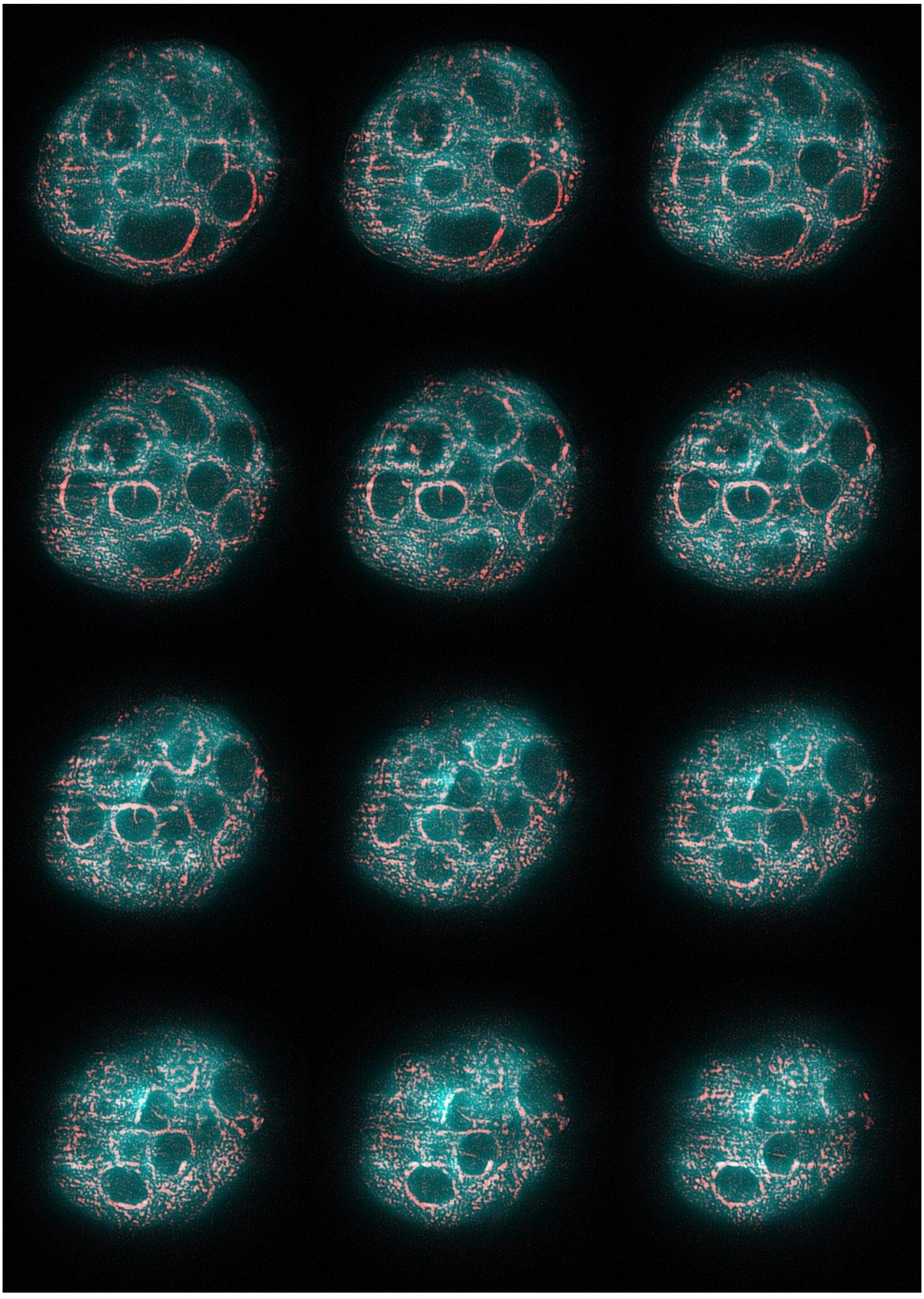

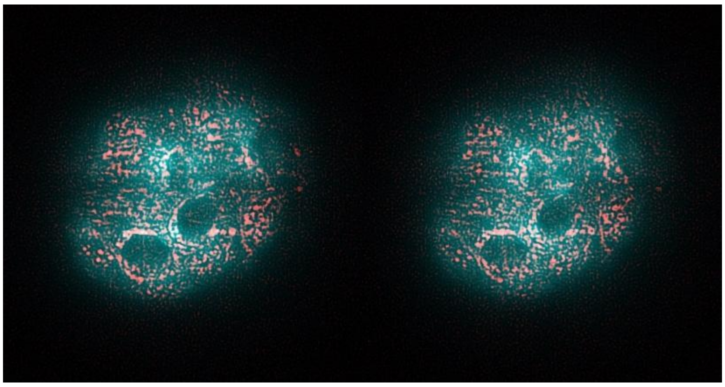

Figure 3 (Supplementary). Stack of 50 out of 70 images with interval of 2 μm along the detection axis obtained with the 4X (XLFLUOR4X/340, NA:0.28, Olympus/Japan) objective.
